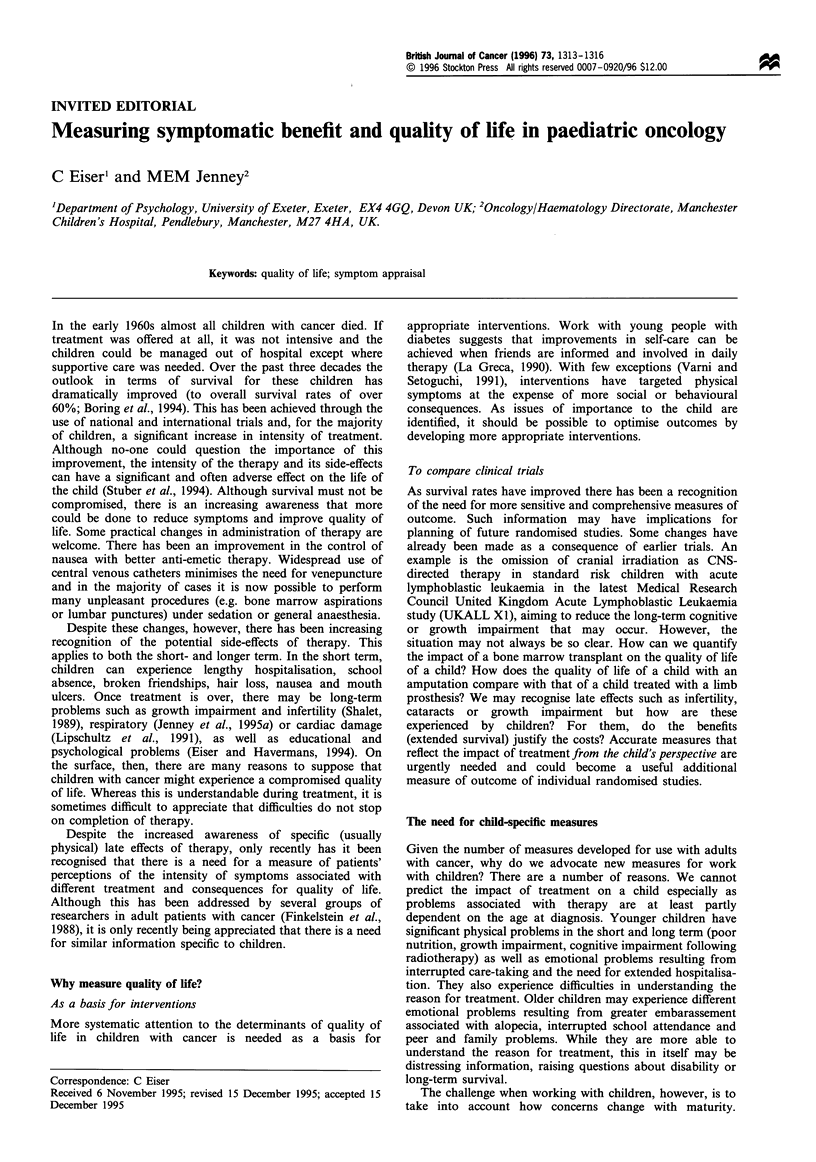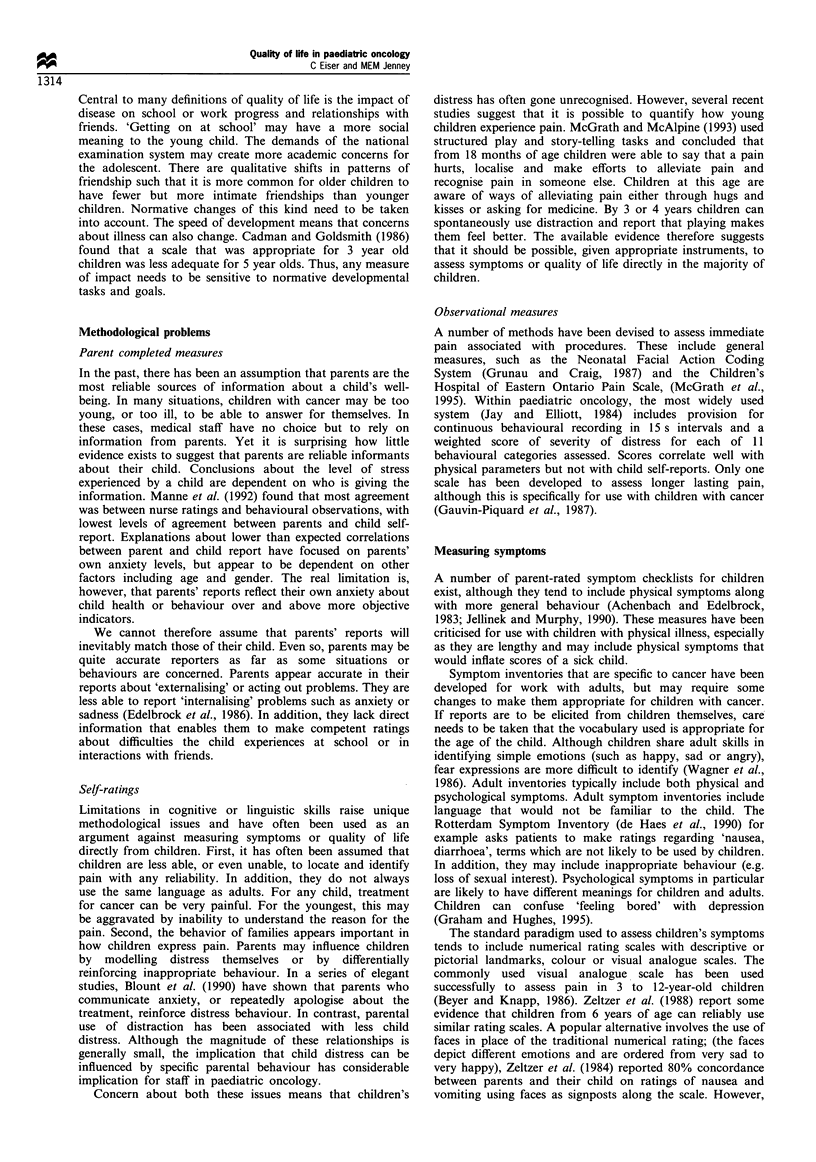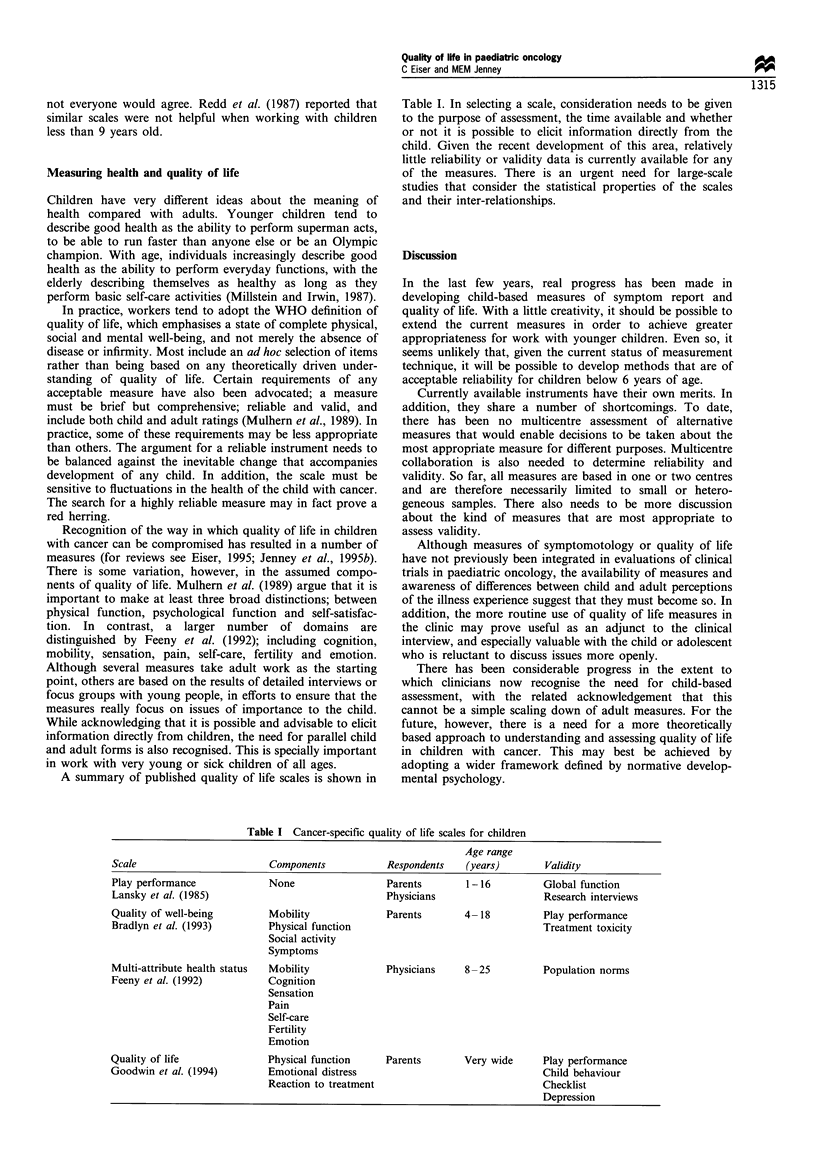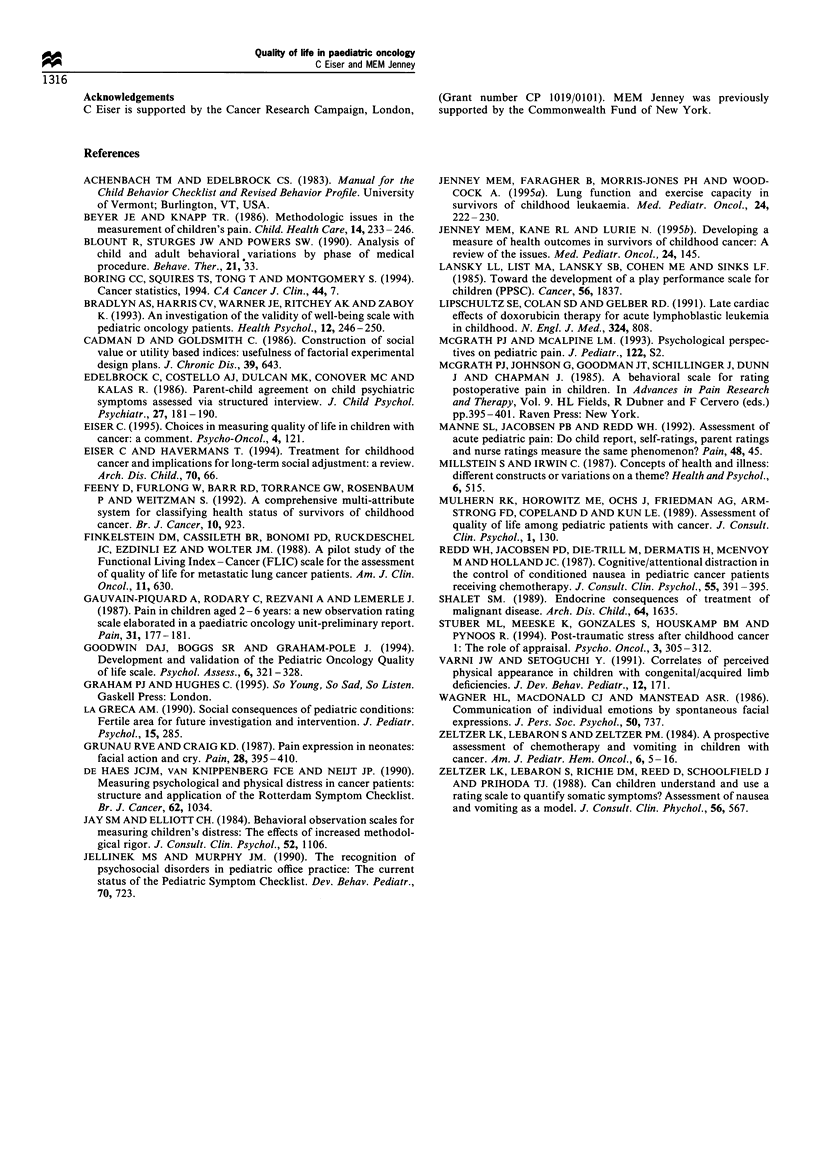# Measuring symptomatic benefit and quality of life in paediatric oncology.

**DOI:** 10.1038/bjc.1996.251

**Published:** 1996-06

**Authors:** C. Eiser, M. E. Jenney


British Journal of Cancer (1996) 73, 1313–1316
© 1996 Stockton Press All rights reserved 0007–0920/96 $12.00

INVITED EDITORIAL

### Measuring symptomatic benefit and quality of life in paediatric oncology

C Eiser[1] and MEM Jenney[2]

[1]*Department of Psychology, University of Exeter, Exeter, EX4 4GQ, Devon UK;* [2]*Oncology/Haematology Directorate, Manchester Children's Hospital, Pendlebury, Manchester, M27 4HA, UK.*

**Keywords:** quality of life; symptom appraisal

In the early 1960s almost all children with cancer died. If treatment was offered at all, it was not intensive and the children could be managed out of hospital except where supportive care was needed. Over the past three decades the outlook in terms of survival for these children has dramatically improved (to overall survival rates of over 60%; Boring *et al.*, 1994). This has been achieved through the use of national and international trials and, for the majority of children, a significant increase in intensity of treatment. Although no-one could question the importance of this improvement, the intensity of the therapy and its side-effects can have a significant and often adverse effect on the life of the child (Stuber *et al.*, 1994). Although survival must not be compromised, there is an increasing awareness that more could be done to reduce symptoms and improve quality of life. Some practical changes in administration of therapy are welcome. There has been an improvement in the control of nausea with better anti-emetic therapy. Widespread use of central venous catheters minimises the need for venepuncture and in the majority of cases it is now possible to perform many unpleasant procedures (e.g. bone marrow aspirations or lumbar punctures) under sedation or general anaesthesia.

Despite these changes, however, there has been increasing recognition of the potential side-effects of therapy. This applies to both the short- and longer term. In the short term, children can experience lengthy hospitalisation, school absence, broken friendships, hair loss, nausea and mouth ulcers. Once treatment is over, there may be long-term problems such as growth impairment and infertility (Shalet, 1989), respiratory (Jenney *et al.*, 1995*a*) or cardiac damage (Lipschultz *et al.*, 1991), as well as educational and psychological problems (Eiser and Havermans, 1994). On the surface, then, there are many reasons to suppose that children with cancer might experience a compromised quality of life. Whereas this is understandable during treatment, it is sometimes difficult to appreciate that difficulties do not stop on completion of therapy.

Despite the increased awareness of specific (usually physical) late effects of therapy, only recently has it been recognised that there is a need for a measure of patients' perceptions of the intensity of symptoms associated with different treatment and consequences for quality of life. Although this has been addressed by several groups of researchers in adult patients with cancer (Finkelstein *et al.*, 1988), it is only recently being appreciated that there is a need for similar information specific to children.

### Why measure quality of life?

### *As a basis for interventions*

More systematic attention to the determinants of quality of life in children with cancer is needed as a basis for appropriate interventions. Work with young people with diabetes suggests that improvements in self-care can be achieved when friends are informed and involved in daily therapy (La Greca, 1990). With few exceptions (Varni and Setoguchi, 1991), interventions have targeted physical symptoms at the expense of more social or behavioural consequences. As issues of importance to the child are identified, it should be possible to optimise outcomes by developing more appropriate interventions.

### *To compare clinical trials*

As survival rates have improved there has been a recognition of the need for more sensitive and comprehensive measures of outcome. Such information may have implications for planning of future randomised studies. Some changes have already been made as a consequence of earlier trials. An example is the omission of cranial irradiation as CNS-directed therapy in standard risk children with acute lymphoblastic leukaemia in the latest Medical Research Council United Kingdom Acute Lymphoblastic Leukaemia study (UKALL X1), aiming to reduce the long-term cognitive or growth impairment that may occur. However, the situation may not always be so clear. How can we quantify the impact of a bone marrow transplant on the quality of life of a child? How does the quality of life of a child with an amputation compare with that of a child treated with a limb prosthesis? We may recognise late effects such as infertility, cataracts or growth impairment but how are these experienced by children? For them, do the benefits (extended survival) justify the costs? Accurate measures that reflect the impact of treatment *from the child's perspective* are urgently needed and could become a useful additional measure of outcome of individual randomised studies.

### The need for child-specific measures

Given the number of measures developed for use with adults with cancer, why do we advocate new measures for work with children? There are a number of reasons. We cannot predict the impact of treatment on a child especially as problems associated with therapy are at least partly dependent on the age at diagnosis. Younger children have significant physical problems in the short and long term (poor nutrition, growth impairment, cognitive impairment following radiotherapy) as well as emotional problems resulting from interrupted care-taking and the need for extended hospitalisation. They also experience difficulties in understanding the reason for treatment. Older children may experience different emotional problems resulting from greater embarassement associated with alopecia, interrupted school attendance and peer and family problems. While they are more able to understand the reason for treatment, this in itself may be distressing information, raising questions about disability or long-term survival.

The challenge when working with children, however, is to take into account how concerns change with maturity.

Correspondence: C Eiser
Received 6 November 1995; revised 15 December 1995; accepted 15 December 1995

Central to many definitions of quality of life is the impact of disease on school or work progress and relationships with friends. 'Getting on at school' may have a more social meaning to the young child. The demands of the national examination system may create more academic concerns for the adolescent. There are qualitative shifts in patterns of friendship such that it is more common for older children to have fewer but more intimate friendships than younger children. Normative changes of this kind need to be taken into account. The speed of development means that concerns about illness can also change. Cadman and Goldsmith (1986) found that a scale that was appropriate for 3 year old children was less adequate for 5 year olds. Thus, any measure of impact needs to be sensitive to normative developmental tasks and goals.

### Methodological problems

### *Parent completed measures*

In the past, there has been an assumption that parents are the most reliable sources of information about a child's well-being. In many situations, children with cancer may be too young, or too ill, to be able to answer for themselves. In these cases, medical staff have no choice but to rely on information from parents. Yet it is surprising how little evidence exists to suggest that parents are reliable informants about their child. Conclusions about the level of stress experienced by a child are dependent on who is giving the information. Manne *et al.* (1992) found that most agreement was between nurse ratings and behavioural observations, with lowest levels of agreement between parents and child self-report. Explanations about lower than expected correlations between parent and child report have focused on parents' own anxiety levels, but appear to be dependent on other factors including age and gender. The real limitation is, however, that parents' reports reflect their own anxiety about child health or behaviour over and above more objective indicators.

We cannot therefore assume that parents' reports will inevitably match those of their child. Even so, parents may be quite accurate reporters as far as some situations or behaviours are concerned. Parents appear accurate in their reports about 'externalising' or acting out problems. They are less able to report 'internalising' problems such as anxiety or sadness (Edelbrock *et al.*, 1986). In addition, they lack direct information that enables them to make competent ratings about difficulties the child experiences at school or in interactions with friends.

### *Self-ratings*

Limitations in cognitive or linguistic skills raise unique methodological issues and have often been used as an argument against measuring symptoms or quality of life directly from children. First, it has often been assumed that children are less able, or even unable, to locate and identify pain with any reliability. In addition, they do not always use the same language as adults. For any child, treatment for cancer can be very painful. For the youngest, this may be aggravated by inability to understand the reason for the pain. Second, the behavior of families appears important in how children express pain. Parents may influence children by modelling distress themselves or by differentially reinforcing inappropriate behaviour. In a series of elegant studies, Blount *et al.* (1990) have shown that parents who communicate anxiety, or repeatedly apologise about the treatment, reinforce distress behaviour. In contrast, parental use of distraction has been associated with less child distress. Although the magnitude of these relationships is generally small, the implication that child distress can be influenced by specific parental behaviour has considerable implication for staff in paediatric oncology.

Concern about both these issues means that children's distress has often gone unrecognised. However, several recent studies suggest that it is possible to quantify how young children experience pain. McGrath and McAlpine (1993) used structured play and story-telling tasks and concluded that from 18 months of age children were able to say that a pain hurts, localise and make efforts to alleviate pain and recognise pain in someone else. Children at this age are aware of ways of alleviating pain either through hugs and kisses or asking for medicine. By 3 or 4 years children can spontaneously use distraction and report that playing makes them feel better. The available evidence therefore suggests that it should be possible, given appropriate instruments, to assess symptoms or quality of life directly in the majority of children.

### *Observational measures*

A number of methods have been devised to assess immediate pain associated with procedures. These include general measures, such as the Neonatal Facial Action Coding System (Grunau and Craig, 1987) and the Children's Hospital of Eastern Ontario Pain Scale, (McGrath *et al.*, 1995). Within paediatric oncology, the most widely used system (Jay and Elliott, 1984) includes provision for continuous behavioural recording in 15 s intervals and a weighted score of severity of distress for each of 11 behavioural categories assessed. Scores correlate well with physical parameters but not with child self-reports. Only one scale has been developed to assess longer lasting pain, although this is specifically for use with children with cancer (Gauvin-Piquard *et al.*, 1987).

### Measuring symptoms

A number of parent-rated symptom checklists for children exist, although they tend to include physical symptoms along with more general behaviour (Achenbach and Edelbrock, 1983; Jellinek and Murphy, 1990). These measures have been criticised for use with children with physical illness, especially as they are lengthy and may include physical symptoms that would inflate scores of a sick child.

Symptom inventories that are specific to cancer have been developed for work with adults, but may require some changes to make them appropriate for children with cancer. If reports are to be elicited from children themselves, care needs to be taken that the vocabulary used is appropriate for the age of the child. Although children share adult skills in identifying simple emotions (such as happy, sad or angry), fear expressions are more difficult to identify (Wagner *et al.*, 1986). Adult inventories typically include both physical and psychological symptoms. Adult symptom inventories include language that would not be familiar to the child. The Rotterdam Symptom Inventory (de Haes *et al.*, 1990) for example asks patients to make ratings regarding 'nausea, diarrhoea', terms which are not likely to be used by children. In addition, they may include inappropriate behaviour (e.g. loss of sexual interest). Psychological symptoms in particular are likely to have different meanings for children and adults. Children can confuse 'feeling bored' with depression (Graham and Hughes, 1995).

The standard paradigm used to assess children's symptoms tends to include numerical rating scales with descriptive or pictorial landmarks, colour or visual analogue scales. The commonly used visual analogue scale has been used successfully to assess pain in 3 to 12-year-old children (Beyer and Knapp, 1986). Zeltzer *et al.* (1988) report some evidence that children from 6 years of age can reliably use similar rating scales. A popular alternative involves the use of faces in place of the traditional numerical rating; (the faces depict different emotions and are ordered from very sad to very happy), Zeltzer *et al.* (1984) reported 80% concordance between parents and their child on ratings of nausea and vomiting using faces as signposts along the scale. However, not everyone would agree. Redd *et al.* (1987) reported that similar scales were not helpful when working with children less than 9 years old.

### Measuring health and quality of life

Children have very different ideas about the meaning of health compared with adults. Younger children tend to describe good health as the ability to perform superman acts, to be able to run faster than anyone else or be an Olympic champion. With age, individuals increasingly describe good health as the ability to perform everyday functions, with the elderly describing themselves as healthy as long as they perform basic self-care activities (Millstein and Irwin, 1987).

In practice, workers tend to adopt the WHO definition of quality of life, which emphasises a state of complete physical, social and mental well-being, and not merely the absence of disease or infirmity. Most include an *ad hoc* selection of items rather than being based on any theoretically driven understanding of quality of life. Certain requirements of any acceptable measure have also been advocated; a measure must be brief but comprehensive; reliable and valid, and include both child and adult ratings (Mulhern *et al.*, 1989). In practice, some of these requirements may be less appropriate than others. The argument for a reliable instrument needs to be balanced against the inevitable change that accompanies development of any child. In addition, the scale must be sensitive to fluctuations in the health of the child with cancer. The search for a highly reliable measure may in fact prove a red herring.

Recognition of the way in which quality of life in children with cancer can be compromised has resulted in a number of measures (for reviews see Eiser, 1995; Jenney *et al.*, 1995*b*). There is some variation, however, in the assumed components of quality of life. Mulhern *et al.* (1989) argue that it is important to make at least three broad distinctions; between physical function, psychological function and self-satisfaction. In contrast, a larger number of domains are distinguished by Feeny *et al.* (1992); including cognition, mobility, sensation, pain, self-care, fertility and emotion. Although several measures take adult work as the starting point, others are based on the results of detailed interviews or focus groups with young people, in efforts to ensure that the measures really focus on issues of importance to the child. While acknowledging that it is possible and advisable to elicit information directly from children, the need for parallel child and adult forms is also recognised. This is specially important in work with very young or sick children of all ages.

A summary of published quality of life scales is shown in Table I. In selecting a scale, consideration needs to be given to the purpose of assessment, the time available and whether or not it is possible to elicit information directly from the child. Given the recent development of this area, relatively little reliability or validity data is currently available for any of the measures. There is an urgent need for large-scale studies that consider the statistical properties of the scales and their inter-relationships.

### Discussion

In the last few years, real progress has been made in developing child-based measures of symptom report and quality of life. With a little creativity, it should be possible to extend the current measures in order to achieve greater appropriateness for work with younger children. Even so, it seems unlikely that, given the current status of measurement technique, it will be possible to develop methods that are of acceptable reliability for children below 6 years of age.

Currently available instruments have their own merits. In addition, they share a number of shortcomings. To date, there has been no multicentre assessment of alternative measures that would enable decisions to be taken about the most appropriate measure for different purposes. Multicentre collaboration is also needed to determine reliability and validity. So far, all measures are based in one or two centres and are therefore necessarily limited to small or heterogeneous samples. There also needs to be more discussion about the kind of measures that are most appropriate to assess validity.

Although measures of symptomotology or quality of life have not previously been integrated in evaluations of clinical trials in paediatric oncology, the availability of measures and awareness of differences between child and adult perceptions of the illness experience suggest that they must become so. In addition, the more routine use of quality of life measures in the clinic may prove useful as an adjunct to the clinical interview, and especially valuable with the child or adolescent who is reluctant to discuss issues more openly.

There has been considerable progress in the extent to which clinicians now recognise the need for child-based assessment, with the related acknowledgement that this cannot be a simple scaling down of adult measures. For the future, however, there is a need for a more theoretically based approach to understanding and assessing quality of life in children with cancer. This may best be achieved by adopting a wider framework defined by normative developmental psychology.

**Table I** Cancer-specific quality of life scales for children

| *Scale* | *Components* | *Respondents* | *Age range (years)* | *Validity* |
|---|---|---|---|---|
| Play performance<br>Lansky *et al.* (1985) | None | Parents<br>Physicians | 1–16 | Global function<br>Research interviews |
| Quality of well-being<br>Bradlyn *et al.* (1993) | Mobility<br>Physical function<br>Social activity<br>Symptoms | Parents | 4–18 | Play performance<br>Treatment toxicity |
| Multi-attribute health status<br>Feeny *et al.* (1992) | Mobility<br>Cognition<br>Sensation<br>Pain<br>Self-care<br>Fertility<br>Emotion | Physicians | 8–25 | Population norms |
| Quality of life<br>Goodwin *et al.* (1994) | Physical function<br>Emotional distress<br>Reaction to treatment | Parents | Very wide | Play performance<br>Child behaviour<br>Checklist<br>Depression |

**Acknowledgements**
C Eiser is supported by the Cancer Research Campaign, London, (Grant number CP 1019/0101). MEM Jenney was previously supported by the Commonwealth Fund of New York.

### References

ACHENBACH TM AND EDELBROCK CS. (1983). *Manual for the Child Behavior Checklist and Revised Behavior Profile.* University of Vermont; Burlington, VT, USA.

BEYER JE AND KNAPP TR. (1986). Methodologic issues in the measurement of children's pain. *Child. Health Care*, **14,** 233–246.

BLOUNT R, STURGES JW AND POWERS SW. (1990). Analysis of child and adult behavioral variations by phase of medical procedure. *Behave. Ther.*, **21,** 33.

BORING CC, SQUIRES TS, TONG T AND MONTGOMERY S. (1994). Cancer statistics, 1994. *CA Cancer J. Clin.*, **44,** 7.

BRADLYN AS, HARRIS CV, WARNER JE, RITCHEY AK AND ZABOY K. (1993). An investigation of the validity of well-being scale with pediatric oncology patients. *Health Psychol.*, **12,** 246–250.

CADMAN D AND GOLDSMITH C. (1986). Construction of social value or utility based indices: usefulness of factorial experimental design plans. *J. Chronic Dis.*, **39,** 643.

EDELBROCK C, COSTELLO AJ, DULCAN MK, CONOVER MC AND KALAS R. (1986). Parent-child agreement on child psychiatric symptoms assessed via structured interview. *J. Child Psychol. Psychiatr.*, **27,** 181–190.

EISER C. (1995). Choices in measuring quality of life in children with cancer: a comment. *Psycho-Oncol.*, **4,** 121.

EISER C AND HAVERMANS T. (1994). Treatment for childhood cancer and implications for long-term social adjustment: a review. *Arch. Dis. Child.*, **70,** 66.

FEENY D, FURLONG W, BARR RD, TORRANCE GW, ROSENBAUM P AND WEITZMAN S. (1992). A comprehensive multi-attribute system for classifying health status of survivors of childhood cancer. *Br. J. Cancer*, **10,** 923.

FINKELSTEIN DM, CASSILETH BR, BONOMI PD, RUCKDESCHEL JC, EZDINLI EZ AND WOLTER JM. (1988). A pilot study of the Functional Living Index – Cancer (FLIC) scale for the assessment of quality of life for metastatic lung cancer patients. *Am. J. Clin. Oncol.*, **11,** 630.

GAUVAIN-PIQUARD A, RODARY C, REZVANI A AND LEMERLE J. (1987). Pain in children aged 2–6 years: a new observation rating scale elaborated in a paediatric oncology unit-preliminary report. *Pain*, **31,** 177–181.

GOODWIN DAJ, BOGGS SR AND GRAHAM-POLE J. (1994). Development and validation of the Pediatric Oncology Quality of life scale. *Psychol. Assess.*, **6,** 321–328.

GRAHAM PJ AND HUGHES C. (1995). *So Young, So Sad, So Listen.* Gaskell Press: London.

LA GRECA AM. (1990). Social consequences of pediatric conditions: Fertile area for future investigation and intervention. *J. Pediatr. Psychol.*, **15,** 285.

GRUNAU RVE AND CRAIG KD. (1987). Pain expression in neonates: facial action and cry. *Pain*, **28,** 395–410.

DE HAES JCJM, VAN KNIPPENBERG FCE AND NEIJT JP. (1990). Measuring psychological and physical distress in cancer patients: structure and application of the Rotterdam Symptom Checklist. *Br. J. Cancer*, **62,** 1034.

JAY SM AND ELLIOTT CH. (1984). Behavioral observation scales for measuring children's distress: The effects of increased methodolgical rigor. *J. Consult. Clin. Psychol.*, **52,** 1106.

JELLINEK MS AND MURPHY JM. (1990). The recognition of psychosocial disorders in pediatric office practice: The current status of the Pediatric Symptom Checklist. *Dev. Behav. Pediatr.*, **70,** 723.

JENNEY MEM, FARAGHER B, MORRIS-JONES PH AND WOODCOCK A. (1995*a*). Lung function and exercise capacity in survivors of childhood leukaemia. *Med. Pediatr. Oncol.*, **24,** 222–230.

JENNEY MEM, KANE RL AND LURIE N. (1995*b*). Developing a measure of health outcomes in survivors of childhood cancer: A review of the issues. *Med. Pediatr. Oncol.*, **24,** 145.

LANSKY LL, LIST MA, LANSKY SB, COHEN ME AND SINKS LF. (1985). Toward the development of a play performance scale for children (PPSC). *Cancer*, **56,** 1837.

LIPSCHULTZ SE, COLAN SD AND GELBER RD. (1991). Late cardiac effects of doxorubicin therapy for acute lymphoblastic leukemia in childhood. *N. Engl. J. Med.*, **324,** 808.

MCGRATH PJ AND MCALPINE LM. (1993). Psychological perspectives on pediatric pain. *J. Pediatr.*, **122,** S2.

MCGRATH PJ, JOHNSON G, GOODMAN JT, SCHILLINGER J, DUNN J AND CHAPMAN J. (1985). A behavioral scale for rating postoperative pain in children. In *Advances in Pain Research and Therapy*, Vol. 9. HL Fields, R Dubner and F Cervero (eds.) pp.395–401. Raven Press: New York.

MANNE SL, JACOBSEN PB AND REDD WH. (1992). Assessment of acute pediatric pain: Do child report, self-ratings, parent ratings and nurse ratings measure the same phenomenon? *Pain*, **48,** 45.

MILLSTEIN S AND IRWIN C. (1987). Concepts of health and illness: different constructs or variations on a theme? *Health and Psychol.*, **6,** 515.

MULHERN RK, HOROWITZ ME, OCHS J, FRIEDMAN AG, ARMSTRONG FD, COPELAND D AND KUN LE. (1989). Assessment of quality of life among pediatric patients with cancer. *J. Consult. Clin. Psychol.*, **1,** 130.

REDD WH, JACOBSEN PD, DIE-TRILL M, DERMATIS H, MCENVOY M AND HOLLAND JC. (1987). Cognitive/attentional distraction in the control of conditioned nausea in pediatric cancer patients receiving chemotherapy. *J. Consult. Clin. Psychol.*, **55,** 391–395.

SHALET SM. (1989). Endocrine consequences of treatment of malignant disease. *Arch. Dis. Child.*, **64,** 1635.

STUBER ML, MEESKE K, GONZALES S, HOUSKAMP BM AND PYNOOS R. (1994). Post-traumatic stress after childhood cancer 1: The role of appraisal. *Psycho. Oncol.*, **3,** 305–312.

VARNI JW AND SETOGUCHI Y. (1991). Correlates of perceived physical appearance in children with congenital/acquired limb deficiencies. *J. Dev. Behav. Pediatr.*, **12,** 171.

WAGNER HL, MACDONALD CJ AND MANSTEAD ASR. (1986). Communication of individual emotions by spontaneous facial expressions. *J. Pers. Soc. Psychol.*, **50,** 737.

ZELTZER LK, LEBARON S AND ZELTZER PM. (1984). A prospective assessment of chemotherapy and vomiting in children with cancer. *Am. J. Pediatr. Hem. Oncol.*, **6,** 5–16.

ZELTZER LK, LEBARON S, RICHIE DM, REED D, SCHOOLFIELD J AND PRIHODA TJ. (1988). Can children understand and use a rating scale to quantify somatic symptoms? Assessment of nausea and vomiting as a model. *J. Consult. Clin. Phychol.*, **56,** 567.